# A Model for Estimating the Risk of Gastrointestinal Bleeding for Patients on Oral Anticoagulants and Interacting Medications

**DOI:** 10.1002/phar.70205

**Published:** 2026-10-25

**Authors:** Ainhoa Gomez‐Lumbreras, Abdelrahman G. Tawfik, Thomas Reese, Daniel M. Witt, Guilherme Del Fiol, Daniel C. Malone

**Affiliations:** ^1^ Department of Pharmacotherapy College of Pharmacy, University of Utah Salt Lake City Utah USA; ^2^ Department of Biomedical Informatics Vanderbilt University Medical Center Nashville Tennessee USA; ^3^ Department of Biomedical Informatics, School of Medicine University of Utah Salt Lake City Utah USA

**Keywords:** anticoagulants, clinical, decision support systems, drug interactions, gastrointestinal hemorrhage, risk assessment

## Abstract

**Introduction:**

Pharmacodynamic drug–drug interactions (DDI) in patients receiving oral anticoagulants (OAC) may increase the risk of gastrointestinal bleeding (GIB). The purpose of this study was to develop and validate a GIB model for patients receiving OACs.

**Methods:**

A risk model, **D**rug **I**nteractions in **O**ral **A**nti**C**oagulants for Risk of **G**astro**i**ntestinal **B**leeding (DIOAC‐GIB), was developed to predict GIB based on interacting medications and other patient‐specific risk factors for patients on OAC. Inputs for the model were obtained using random‐effect meta‐analysis or from the literature for the following attributes: exposure to nonsteroidal anti‐inflammatory drugs (NSAIDs); selective serotonin reuptake inhibitors/serotonin‐norepinephrine reuptake inhibitors (SSRI/SNRIs); aspirin, antiplatelet medications, and proton pump inhibitors (PPIs); age; and GIB history. The model was correlated to existing OAC models (HAS‐BLED and DOAC score).

**Results:**

GIB risk for OACs was based on pivotal trials (1.16% for apixaban; 3.15% for rivaroxaban; 3.00% for dabigatran; 3.31% for edoxaban; and 2.00% for warfarin). The odds ratio (OR) of GIB for NSAIDs ranged from 1.16 (95% confidence interval [CI]: 0.84–1.61) for celecoxib to 20.67 (95% CI: 14.56–29.34) for ketorolac. ORs for GIB among SSRI/SNRIs ranged from 1.31 (95% CI: 1.07–1.62) for paroxetine to 1.50 (95% CI: 1.32–1.70) for venlafaxine. Risk of GIB also included persons > 65 years of age (relative risk = 2.50, 95% CI: 1.2–5.5); history of GIB (OR = 5.14, 95% CI: 3.92–6.73); exposure to aspirin (OR = 1.31, 95% CI: 0.93–1.85), antiplatelet medications (OR = 1.97, 95% CI: 1.73–2.25), and corticosteroids (OR = 1.69, 95% CI: 1.53–1.87); and PPIs reduced GIB risk (OR = 0.67, 95% CI: 0.62–0.74). Correlations between the DIOAC‐GIB and HAS‐BLED and DOAC scores were 0.5 for both (*p* < 0.01).

**Conclusion:**

DIOAC‐GIB is a novel GIB‐risk model that incorporates pharmacodynamic OAC DDIs to assist clinicians and patients to determine a therapeutic plan while accounting for GIB risk. DIOAC‐GIB correlates with other bleeding risk scores and may help identify high‐risk patients who may benefit from tailored prescribing.

## 1 Introduction

There is little support for clinicians when treating complex patients receiving oral anticoagulants (OAC) [1]. OACs are frequently implicated in pharmacodynamic drug–drug interactions (DDIs) because of their propensity to lead to bleeding episodes, especially gastrointestinal bleeding (GIB) events [2, 3]. OACs include warfarin (a vitamin K antagonist), and four other OACs (i.e., apixaban, dabigatran, edoxaban, rivaroxaban) called direct‐acting OAC (DOAC) that affect the clotting cascade [4]. Patients taking OAC tend to be older and often have multiple comorbidities requiring numerous medications that increase the risk of bleeding due to DDIs [5, 6, 7]. Anticoagulation guidelines recommend evaluating the patient's risk for bleeding at the initiation of OAC [8]. Studies have shown that patients taking concurrent warfarin and non‐steroidal anti‐inflammatory drugs (NSAIDs) have approximately a 2‐fold increase in the risk of GIB [9].

Major bleeding events when using warfarin have been estimated at up to 7.2 per 100 persons‐year, with the gastrointestinal tract being the most common site of bleeding [10]. Thirty‐day mortality from a major GIB is approximately 8% in patients receiving DOACs [11]. Several bleeding risk prediction models have been developed to help clinicians assess the risk of major bleeding for patients on anticoagulants [12, 13, 14, 15, 16, 17, 18]. The HAS‐BLED score is one risk calculator for patients with atrial fibrillation (AF) who are receiving warfarin [12]. It includes factors such as uncontrolled hypertension, abnormal liver or renal function, stroke history, prior bleeding, labile international normalized ratio (INR), advanced age (over 65 years), and medications (one point increase for use of aspirin, NSAIDs, or clopidogrel) and alcohol use. However, HAS‐BLED only provides a single point for the use of medications like NSAIDs and antiplatelet medications and entirely omits the bleeding risk from other medications, including selective serotonin reuptake inhibitors (SSRIs), serotonin‐norepinephrine reuptake inhibitors (SNRIs), and oral corticosteroids. Furthermore, most of these risk factors are non‐modifiable. Another tool is the DOAC score, a model developed specifically for patients prescribed DOACs and includes risk factors such as renal function, age, and NSAID use [13]. However, like HAS‐BLED, the DOAC score does not differentiate between individual NSAID or SSRI medications despite their known variability in GIB risk. Other models include the ORBIT score [19] and ATRIA [20]. These risk models have several limitations, including the lack of specificity with respect to medications, often grouping different therapeutic classes together and/or not accounting for differences in GIB between products within a class (e.g., celecoxib vs. ketorolac) [21].

The purposes of this study were to: (i) create a model to predict GIB among OAC users using medications that interact pharmacodynamically with OACs, especially NSAIDs and SSRI/SNRIs, and (ii) evaluate criterion‐related validity of the model with HAS‐BLED and DOAC scores.

## 2 Methods

The approach we used in this analysis consisted of two steps: (i) develop a medication‐specific model to assess the risk of GIB among patients taking OACs, and (ii) validate the model against widely used bleeding risk scores. The synthesis model was constructed using disparate evidence of GIB risk for medications and known risk factors for GIB, including age and history of GIB. Synthesis models are similar to decision models in that they combine evidence from multiple sources to reach a conclusion about the benefits, harms, or costs of a given treatment strategy [22]. For this model we employed a logistic risk model that combined univariate risk estimates using log‐odds to predict the probability of a GIB event among OAC users. A de novo risk model called **D**rug **I**nteractions in **O**ral **A**nti**C**oagulants for Risk of **G**astro**i**ntestinal **B**leeding (DIOAC‐GIB) was developed and calibrated based on pivotal trials for the DOACs and a meta‐analysis for GIB risk for warfarin.

### 2.1 Selection of GIB Risk Factors

Experts in DDIs, health outcomes, and clinical decision support were assembled to develop algorithms that could be implemented in electronic health records (EHR) to identify clinically relevant DDIs for a subset of medications that included the combination of OAC and NSAIDs and/or SSRI/SNRIs [23]. Meetings were held virtually to discuss risk factors and the evidence associated with exposure to the interaction and other known risk factors. Experts determined that the following conditions and medications are relevant to estimate the risk of GIB: age > 65 years; previous history of GIB; antiplatelet medications; aspirin; corticosteroids; proton pump inhibitors (PPIs); NSAIDs; and SSRI/SNRIs. For each risk factor identified by the expert panel, estimates of risk were obtained from published literature. Searches of published articles were conducted on PubMed using GIB as a MeSH term (“Gastrointestinal Hemorrhage”) and as free text combined with each risk factor and/or medication of interest (see Table S1). No restrictions were placed on the search criteria. Citations available in PubMed from inception through June 2025 were evaluated for inclusion. Furthermore, medication compendia publishing DDIs were evaluated (i.e., Lexicomp, Micromedex, Drugs.com) for references and evidence regarding the selected medications and attributes of interest [24, 25, 26]. Cited studies and meta‐analysis on GIB risk factors were also used to identify potential studies.

### 2.2 Study Selection and Data Extraction

For each risk factor the inclusion criteria for studies of interest were: (i) reported GIB risk regardless of the OAC indication (e.g., non‐valvular AF, valve replacement, deep vein thrombosis, etc.) to increase generalizability; (ii) had a control group that included either OAC monotherapy or placebo; and (iii) reported the risk of GIB using odds ratio (OR), hazard ratios (HR), or risk ratio (RR) or contained sufficient details to calculate the OR or RR. Articles were excluded if: (i) the site of bleeding was not specified and/or (ii) the study design consisted of case reports, case series, and non‐quantitative reviews.

Once studies were identified, an evidence‐based pyramid was used to estimate the risk of GIB for each factor [27]. Published systematic reviews and meta‐analyses were prioritized as the primary sources for risk assessment when available, followed by randomized controlled trials (RCTs) and then observational evidence. Moreover, studies involving an OAC control group were given preference because estimates would account for the increased risk of GIB associated with OACs. When multiple time windows of exposure were reported, data for the first 30 days or less were selected because the model was intended to represent the risk at the initiation of therapy. This assumption is supported by the literature that indicates the risk of GIB declines over time [28].

### 2.3 Model Development Approach

For each risk factor in the model where a meta‐analysis did not exist, we combined the existing evidence using standard meta‐analysis procedures. A random‐effects model was employed to incorporate between‐study heterogeneity. Each factor's risk was transformed to log‐odds and then included in a linear prediction model to estimate the log‐odds risk for a given set of risk factors. The linear prediction of risk was then converted to the probability of GIB using the conventional transformation. The model framework is consistent with decision modeling approaches that incorporate multiple attributes to predict health outcomes [29]. The results of the risk model were expressed as integer values per 100 treated patients. The model was calibrated to model the risk of GIB for each OAC based on the pivotal studies and/or meta‐analyses of risk. This approach is similar to what is used for calibration of cancer simulation models [30]. The specific model equation is shown below:

$$\begin{array}{c} \begin{array}{c} \mathrm{Yi}=\text{−4.67+}0.691^{*}\text{warf}+0.144^{*}\text{apix}+1.147^{*}\text{riva}+1.097^{*}\text{dabi}\\ \;+1.197^{*}\text{edox}+0.916^{*}\mathrm{age}+0.270^{*}\mathrm{asa}+0.678^{*}\text{antiplat}\\ \;+0.523^{*}\text{cortico}+1.637^{*}\text{gibleed}+0.0^{*}\text{buprop}+0.31{\text{9}}^{*}\text{citalo}\\ \;+0.407^{*}\text{desvenlafax}+0.279^{*}\text{duloxe}+0.317^{*}\text{escita}+0.321^{*}\text{fluoxe}\\ \;+0.285^{*}\text{fluvoxa}+0.135^{*}\text{mirta}+0.272^{*}\text{paroxe}+0.291^{*}\text{sertra}\\ \;+0.407^{*}\text{venlafax}+0.149^{*}\text{cele}+1.229^{*}\text{diclo}+0.270^{*}\text{diflunis}\\ \;+1.708^{*}\text{etodolac}+1.461^{*}\text{fenopr}+0.822^{*}\text{ibup}+1.708^{*}\text{indo}\\ \;+1.577^{*}\text{ketoprof}+3.029^{*}\text{ketoro}+0.270^{*}\text{mefen}+1.924^{*}\text{melo}\\ \;+0.270^{*}\text{nabum}+1.461^{*}\text{napr}+1.461^{*}\text{oxapro}+2.223^{*}\text{piro}\\ \;+1.708^{*}\text{sulin}+1.708^{*}\text{tolm}+\;\left(-0.400\right)*\mathrm{ppi}. \end{array} \end{array}$$

where Yi = Log‐odds of GIB and all other risk factors are binary for absence/presence of risk factor

$$\text{Probability of}\;\mathrm{GIB}=\exp^{\mathrm{Yi}}/\left(1+\exp^{\mathrm{Yi}}\right).$$

It is important to keep in mind that this model takes into account the active medications being used by the patient but does not attempt to address bridging therapy for individuals receiving other anticoagulants prior to starting an OAC.

### 2.4 Model Validation

To evaluate the criterion‐related validity of the prediction tool using the HAS‐BLED and DOAC Score, we used a cohort drawn from the University of Utah EPIC EHR system. Patients included in this study were adults (aged ≥ 18 years) with at least one active prescription for an OAC, either warfarin or a DOAC, during the 12‐month window from December 1, 2023, to November 30, 2024. A prevalent‐user design was used, meaning patients may have initiated OAC therapy before the study period but were required to have the medication included in the “current medications” section of the EHR during the timeframe. We excluded patients with missing data for key variables (e.g., age, comorbidities) or overlapping use of both warfarin and/or DOACs within the study period. The HAS‐BLED score required the identification of uncontrolled hypertension, defined as a systolic blood pressure (SBP) > 160 mm Hg [12]. However, SBP values were not reliably available. To address this limitation, we imputed uncontrolled hypertension by randomly assigning 10% of patients with diagnosed hypertension as having “uncontrolled” hypertension. This estimate is supported by national data indicating that approximately 15% of adults in the United States receiving treatment for hypertension continue to have SBP ≥ 130 mm Hg [31]. Assuming 10% of patients exceeding the 160 mm Hg threshold was felt to be both reasonable and conservative in this context.

Patient characteristics and medication exposures were summarized using descriptive statistics. For each patient, we calculated the maximum score during the year, assuming that all prescribed medications were concurrently used. Each observation was stratified into low‐, moderate‐, and high‐risk categories based on HAS‐BLED and DOAC scores. For HAS‐BLED, risk groups were defined as low (0–1), moderate (2), high (≥ 3), and very high (≥ 5). Similarly, for the DOAC score, risk groups were low (≤ 5), moderate (6–7), and high (≥ 8).

We assessed the correlation between *DIOAC‐GIB* and existing bleeding risk tools (HAS‐BLED and DOAC scores) using Spearman's rank correlation coefficients (ρ) given the ordinal nature of the scores and non‐normal distributions of risk scores. The DIOAC‐GIB score was also categorized into low, medium, and high risk relative to the HAS‐BLED and DOAC risk stratification values.

## 3 Results

The risk estimates included in the model can be found in Table 1. For those estimates not obtained from a pivotal trial or previously published meta‐analysis, the studies included in the meta‐analysis of each of the selected risk factors and their characteristics (study design, settping, timeline, risk estimated, adjustment, GIB definition, sample size, sex, and age) are displayed in Table S2.

**TABLE 1:** Model risk estimates for gastrointestinal bleeding.

Oral anticoagulants	GIB rate		Reference(s)
Warfarin	2.00		[32]
Apixaban	1.16		[33]
Dabigatran	3.00		[34]
Rivaroxaban	3.15		[35]
Edoxaban	3.31		[36]
**Others Risk Factors**	**Odds Ratio for GIB**	**95% Confidence Interval**	
Age	2.50	1.2–5.5	[37]
Aspirin	1.31	0.93–1.85	[38]
History of GIB	5.14	3.92–6.73	[37, 39, 40, 41, 42, 43, 44, 45, 46, 47, 48, 49, 50, 51, 52, 53, 54, 55, 56, 57, 58, 59]
Antiplatelet medications	1.97	1.73–2.25	[39, 40, 41, 42, 43, 44, 45, 46, 47, 60, 61, 62, 63, 64, 65, 66, 67, 68, 69, 70, 71, 72, 73, 74, 75, 76, 77, 78, 79, 80, 81]
Corticosteroids	1.69	1.53–1.87	[40, 41, 46, 47, 48, 49, 50, 59, 61, 62, 64, 66, 67, 69, 71, 73, 74, 75, 76, 77, 81, 82, 83, 84, 85, 86, 87, 88, 89, 90]
Proton Pump Inhibitors	0.67 0.67	0.62–0.74^a^ 0.61–0.74^a^	[91, 92]
**NSAIDs**			
Celecoxib	1.16	0.84–1.61	[93]
Diclofenac	3.42	2.58–4.53	
Ibuprofen	2.28	1.71–3.03	
Indomethacin	5.52	4.43–6.86	
Ketoprofen	4.84	3.05–7.68	
Ketorolac	20.67	14.56–29.34	
Meloxicam	6.85	2.34–20.07	
Naproxen	4.31	3.22–5.77	
Piroxicam	9.24	6.34–13.45	
**SSRIs/SNRIs**			
Citalopram	1.38	1.17–1.62	[94]
Duloxetine	1.32	1.16–1.50	
Escitalopram	1.37	1.12–1.69	
Fluoxetine	1.38	1.26–1.51	
Fluvoxamine	1.33	1.17–1.51	
Mirtazapine	1.14	1.01–1.29	
Paroxetine	1.31	1.07–1.62	
Sertraline	1.34	1.14–1.57	
Venlafaxine	1.50	1.32–1.70	

Abbreviations: GIB, gastrointestinal bleeding; NSAIDs, nonsteroidal anti‐inflammatory drugs; SNRI, serotonin and norepinephrine reuptake inhibitor; SSRI, selective serotonin reuptake inhibitor.

^a^ There are two meta‐analyses reporting risk estimates for proton pump inhibitors; the top number is data from reference [91], and the bottom value is from reference [92].

### 3.1 Oral Anticoagulants

The risk of GIB for each OAC was obtained from the pivotal clinical studies used for product approval and included: ARISTOTLE (apixaban) [33]; RELY (dabigatran) [34]; Rocket AF (rivaroxaban) [35]; and ENGAGE AF‐TIMI (edoxaban) [36]. Apixaban had the lowest rate of GIB among the OACs (105 GIB among 9088 participants receiving apixaban, 1.16%) [33]. Studies evaluating dabigatran and rivaroxaban reported 182 events in 6076 participants [34], and 224 events in 7111 participants [35], 3.0% and 3.15%, respectively. The ENGAGE AF‐TIMI trial studying edoxaban reported a rate of 3.31% (232 events in 7012 participants) [36]. For warfarin, we used a published meta‐analysis, that included the previously used RCTs for the DOAC that included warfarin as the control; the GIB rate for warfarin across these studies was 2% [32]. Characteristics of these trials are shown in Table S2.

### 3.2 NSAIDs

For the different NSAID products frequently used in the United States, we used specific risks of GIB from a previously published meta‐analysis [93]. Among the NSAIDs, ketorolac had the highest risk of GIB (OR = 20.67, 95% CI: 14.56–29.34) while celecoxib had the lowest risk (OR = 1.16, 95% CI: 0.84–1.61). Risks of GIB for each specific NSAID are available in Table 1.

### 3.3 Antidepressants SSRIs / SNRIs

For each agent included in the model, we obtained risk estimates by conducting a meta‐analysis on 20 studies (17 observational studies and 3 RCT) [94]. The risk of GIB was shown to be increased by 31% to 50% depending on the agent [from paroxetine OR = 1.31, 95% CI (1.07–1.62) and duloxetine OR = 1.32, 95% CI (1.16–1.50) to venlafaxine OR = 1.50, 95% CI (1.32–1.70)]. Table 1 shows specific risks of GIB for each antidepressant.

### 3.4 Age

Risk of GIB is known to increase with age, but studies providing data that could be used in the model were limited. The model incorporated estimates from Chen et al. who conducted a cohort study of 401 patients on warfarin in Taiwan and found that the relative risk of GIB was 2.5 (95% CI 1.2–5.5) for patients over 65 years of age as compared to those 65 or younger years of age (Table 1) [37].

### 3.5 History of GIB

The risk of GIB after a previous GIB event is a significant risk factor. The estimated risk of GIB with a previous GIB event was estimated to be 5.14 (95% CI 3.92–6.73), see Figure 1a. This evidence came from 20 observational studies and two post hoc analyses of the ARISTOTLE and ROCKET‐AF clinical trials. See Table S2 for the characteristics of the included studies.

**FIGURE 1:**
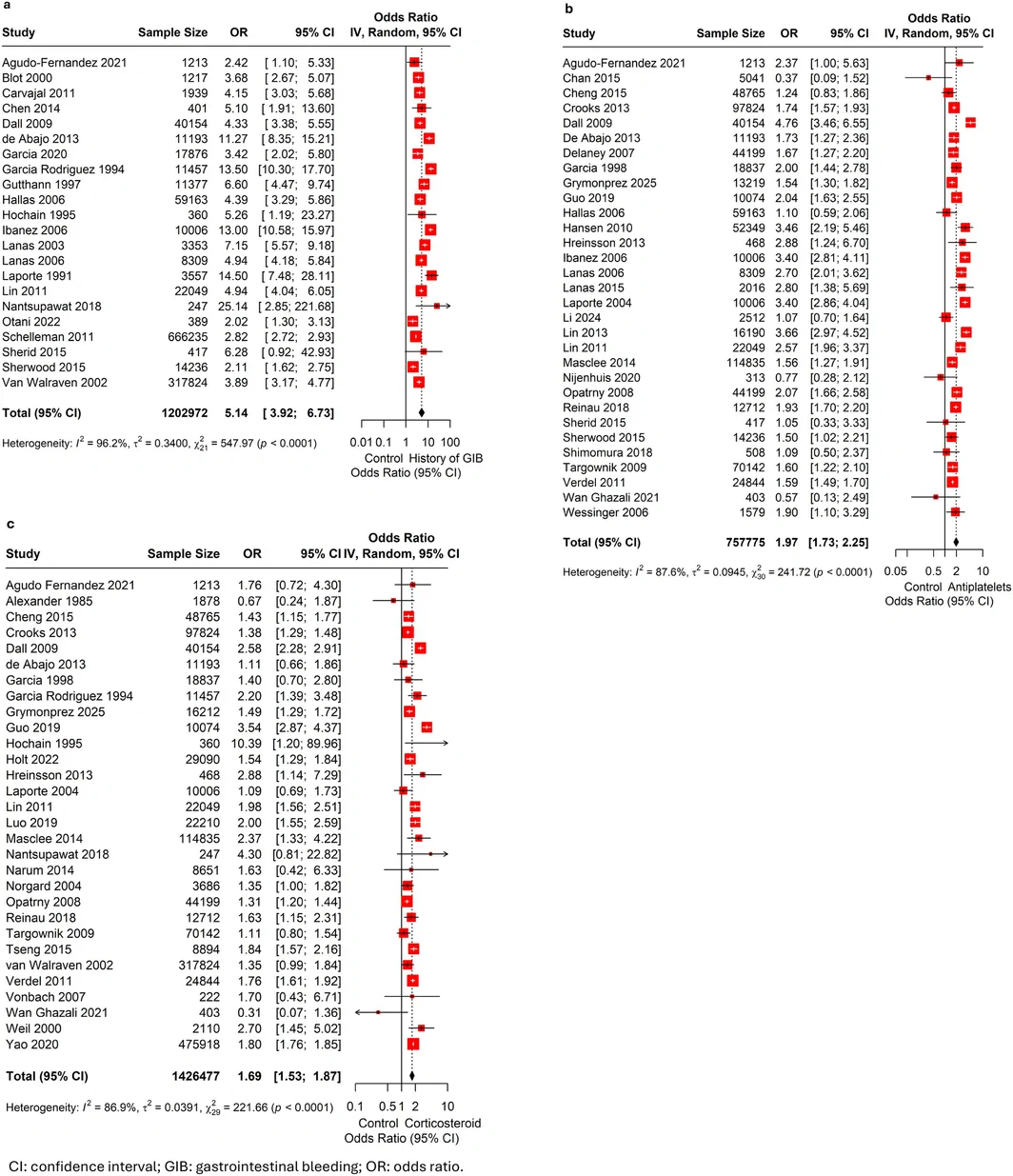
(a) History of Gastrointestinal Bleeding as Risk Factor for Subsequent Gastrointestinal Bleeding.(b) Use of Antiplatelet Medications and Risk of Gastrointestinal Bleeding. (c) Use of Corticosteroids and Risk of Gastrointestinal Bleeding.

### 3.6 Aspirin

The data for the risk of aspirin‐associated GIB was obtained from a systematic review and meta‐analysis by Ghule et al. [38]. This meta‐analysis included studies of patients on a combination of OAC and aspirin compared to OAC only, making it more specific to our risk model, as patients were exposed to a DDI. The OR of GIB for those patients exposed to OAC plus aspirin compared to an OAC alone was 1.31 (95% CI 0.93–1.85).

### 3.7 Antiplatelet Medications

The combined estimate of GIB risk with antiplatelet medications was OR = 1.97 (95% CI: 1.73–2.25), see Figure 1b. This value is based on 31 studies, with most evidence coming from studies that evaluated clopidogrel or grouped all antiplatelet medications together. Studies where the unique effect of clopidogrel and other antiplatelet medications could not be isolated were excluded, as they required participants to be on aspirin, and the treatment groups were dual versus single antiplatelet therapy (aspirin) or short versus long duration of dual antiplatelet therapy [95, 96]. Characteristics of the included studies are shown in Table S2.

### 3.8 Corticosteroids

A total of 30 studies were found to report GIB risk associated with corticosteroids. After combining studies, we estimated the GIB risk to be 1.69 (95% CI 1.53–1.87) as compared to no corticosteroids; see Figure 1c and Table S2 describing the characteristics of all the included studies.

### 3.9 Proton Pump Inhibitors

Based on the mechanism of action, the use of PPI is expected to reduce GIB risk. The estimate of PPI protective effects incorporated data from two comprehensive meta‐analyses [91, 92]. A systematic review and meta‐analysis to evaluate the protective effect of PPI co‐therapy in patients receiving OACs was conducted and included data from 10 studies covering a patient population of 1,970,931 individuals [91]. PPI co‐therapy was associated with a significantly lower odds ratio of any GIB (OR = 0.67; 95% CI: 0.62–0.74) or major GIB (OR = 0.68; 95% CI: 0.63–0.75). Moreover, subgroup analyses showed that the protective effect of PPIs was consistent across different ethnic groups and types of OACs, except edoxaban. Similarly, another meta‐analysis assessed the impact of PPI and histamine 2‐receptor antagonists on the incidence of upper GIB among OAC users [92]. The analysis of five studies concluded that PPIs decreased upper GIB risk (OR = 0.67, 95% CI: 0.61–0.74).

### 3.10 Heterogeneity

The meta‐analysis results for the history of GIB, antiplatelet medications, and corticosteroids showed high heterogeneity (*I* ^2^ > 85%), and to assess potential publication bias, we generated funnel plots (see Figure S1a–c corresponding to the history of GIB, antiplatelet medications, and corticosteroid analyses, respectively). These plots were symmetrical, with most studies within the expected bounds of variation.

### 3.11 Risk Model

After obtaining the estimated GIB risks for each specific attribute or medication, we constructed an evidence‐based GIB risk model. Figure S2 displays an example of how this risk model is displayed through DDInteract, a clinical decision support tool integrated within the EHR to assist clinicians to assess GIB risk and evaluate the risk of various treatment alternatives [97]. The advantage of this tool is that clinicians can explore varying scenarios regarding the use of NSAIDs and/or antidepressants used concurrently with OACs.

### 3.12 Model Validation

From an initial population of 15,905 patients receiving OACs from the University of Utah Enterprise Data Warehouse, 15,357 were retained after excluding those with missing current medication records or diagnoses. A total of 1558 and 9610 patients were included in the warfarin and DOAC cohorts, respectively. Baseline characteristics are shown in Table 2. In both groups, the majority were older than 65 years of age. Notably, GIB history was present in 5.6% of warfarin users compared with 4.3% of DOAC users (*p* = 0.03). Concurrent medication exposure (NSAIDs, SSRI/SNRIs, corticosteroids, and PPIs) was prevalent across both cohorts. NSAID use was more common in patients prescribed DOACs (29.0%) compared to those on warfarin (15.0%) (*p* < 0.0001).

**TABLE 2:** Characteristics of patients on warfarin and Direct‐Acting oral anticoagulants.

Characteristic	Warfarin (*N* = 1558)	DOAC (*N* = 9610)	*p*
Age ≥ 65 years	990 (63.6%)	6050 (62.9%)	0.68
Prior GIB	87 (5.6%)	415 (4.3%)	0.03
Aspirin use	476 (30.6%)	2716 (28.3%)	0.068
Antiplatelet medication use	97 (6.2%)	723 (7.5%)	0.08
Corticosteroid use	316 (20.3%)	2421 (25.2%)	< 0.0001
PPI use	741 (47.6%)	4757 (49.5%)	0.16
NSAID use	233 (15.0%)	2789 (29.0%)	< 0.0001
SSRI/SNRI use	564 (36.2%)	3519 (36.6%)	0.77
Diabetes	97 (6.2%)	742 (7.7%)	0.043
Hypertension	187 (12.0%)	1453 (15.1%)	0.0014
Renal disease	74 (4.8%)	530 (5.5%)	0.28
Stroke history	96 (6.2%)	512 (5.3%)	0.18
Liver disease	35 (2.2%)	231 (2.4%)	0.72
TTR ≥ 60%	985 (63.2%)	—	
Alcohol use > 8 oz/week	32 (2.1%)	—	
GFR < 30 mL/min	—	138 (1.4%)	
GFR 30–60 mL/min	—	1149 (12.0%)	
GFR > 60 mL/min	—	8323 (86.6%)	

Abbreviations: DOAC, direct oral anticoagulant; GFR, glomerular filtration rate; GIB, gastrointestinal bleeding; NSAIDs, nonsteroidal anti‐inflammatory drugs; PPI, proton pump inhibitor; SNRI, serotonin and norepinephrine reuptake inhibitors; SSRI, selective serotonin reuptake inhibitors; TTR, time in therapeutic range.

The mean and standard deviation (SD) GIB risk scores from the DIOAC‐GIB model were 5.48 (SD 5.51) for warfarin users and 4.95 (SD 5.52) for DOAC users, whereas the mean HAS‐BLED (warfarin only) and DOAC scores were 1.65 (SD 0.97) and 3.86 (SD 2.57), respectively (Table 3). The newly derived risk model had moderate but significant correlations with both HAS‐BLED (*r* = 0.50) and DOAC scores (r = 0.49), *p* < 0.01 (Figures S3 and S4).

**TABLE 3:** Distribution of bleeding risk scores.

Statistic	Warfarin		DOAC	
	HAS‐BLED score	DIOAC‐GIB score	DOAC score	DIOAC‐GIB score
Minimum	0.00	1.00	0.00	1.00
25th percentile (Q1)	1.00	3.00	2.00	2.00
75th percentile (Q3)	2.00	6.00	6.00	6.00
Median	2.00	4.00	4.00	3.00
Mean	1.65	5.48	3.86	4.95
Standard Deviation	0.97	5.51	2.57	5.52
Maximum	5.00	87.00	15.00	75.00

Abbreviation: DOAC, direct oral anticoagulant.

Table 4 displays a gradient in DIOAC‐GIB values defined by both HAS‐BLED and DOAC scoring systems. Among patients on warfarin, scores increased steadily from a mean of 3.69 (SD 2.36) in the low‐risk group to 6.08 (SD 5.21) in the moderate‐risk group and 9.06 (SD 8.91) in the high‐risk group. A similar pattern was observed for patients receiving a DOAC, with scores rising from 4.06 (SD 4.41) in low‐risk patients to 6.79 (SD 6.59) in moderate‐risk and 8.89 (SD 8.38) in high‐risk patients. Most patients were classified in the low‐risk group by the DIOAC‐GIB model (62.8% of warfarin users and 67.3% of DOAC users). Moderate‐risk patients represented 21.5% of the warfarin group and 16.2% of the DOAC group, and those classified as high risk accounted for 15.7% and 16.5%, respectively.

**TABLE 4:** Distribution of DIOAC‐GIB scores by HAS‐BLED and DOAC.

Risk tool	Risk group	*N* (%)	DIOAC‐GIB mean (SD)	DIOAC‐GIB median (IQR)	Mean (SD) score for existing models
HAS‐BLED	Low (0–1)	751 (48.20%)	3.69 (2.36)	3 (2–4)	0.81 (0.39)
	Moderate (2)	519 (33.31%)	6.08 (5.21)	4 (3–7)	2.00 (0.00)
	High (≥ 3)	288 (18.48%)	9.06 (8.91)	6 (4–10)	3.19 (0.43)
DOAC	Low (≤ 5)	7102 (73.90%)	4.06 (4.41)	3 (2–5)	2.69 (1.73)
	Moderate (6–7)	1707 (17.76%)	6.79 (6.59)	4 (3–8)	6.40 (0.49)
	High (≥ 8)	801 (8.33%)	8.89 (8.38)	6 (3–11)	8.82 (0.57)

Abbreviations: DOAC, direct oral anticoagulant; IQR, interquartile range; SD, standard deviation.

## 4 Discussion

We developed DIOAC‐GIB to estimate the risk of GIB for patients on OAC accounting for polypharmacy and patient risk factors with an emphasis on modifiable risk factors. Unlike other risk models such as HAS‐BLED and DOAC, which only incorporate broad medication classes, the DIOAC‐GIB uses risk specific to the product for NSAIDs and SSRIs/SNRIs, providing much more granular risk prediction to support clinical decision‐making. The model has been incorporated into DDInteract, an EHR‐integrated app that displays this and other patient view information to assist with clinician‐patient shared decision‐making [97]. A web‐based version of the tool can be found at https://prueba.bmi.utah.edu/ddinteract/. The DDInteract implementation of the DIOAC‐GIB model is unique in that it allows clinicians to evaluate different therapeutic choices for NSAIDs and SSRIs/SNRIs, as well as add or remove other therapies (i.e., aspirin, antiplatelet medications, and PPIs) on GIB risk. This may enhance clinician/patient discussion when considering starting or changing therapy and improve shared decision making [97].

Clinical decision support (CDS) systems aim to guide and prevent harm from DDI [98]. However, alerts for DDIs consider only two medications at a time and contain vague and non‐useful information [99]. Statements about the risk of harm include subjective terms such as: “*minor*”; “*moderate*”; “*serious*”; or “*major*” [24, 25, 100]. Furthermore, warnings do not consider relevant patient attributes and underlying risk factors. More than 90% of the CDS DDI alerts are overridden or ignored due to “*alert fatigue*” and prescribers finding them irrelevant [99, 101, 102, 103]. As a consequence, exposure to life‐threatening DDIs continues to occur despite the widespread use of DDI warnings [104].

The current state of DDI warnings embedded within EHR and pharmacy software is clearly insufficient because those warnings do not display quantifiable information [24, 25, 100, 105]. Although some local improvements on DDI‐CDS have been made, reducing the number of warnings that are overridden remains elusive [106]. For OAC and NSAID or SSRI/SNRI warnings, knowledge vendors (e.g., First DataBank (South San Francisco, CA), Lexicomp (Waltham, MA), MicroMedex (Ann Arbor, MI) etc.) have not historically distinguished between products within a drug class despite wide variability in risk of GIB. Another issue with current warnings is that many patients may benefit from concomitant treatment, especially if medications with a lower risk of bleeding are used or used with gastroprotective medications. Pain is a common reason for seeking medical care, and the availability of over‐the‐counter NSAIDs may suggest to a patient that these medications are safe to use even though the GIB risk is significant [107, 108]. Displaying the risk of combination therapy as a graphic has been shown to increase clarity about the risk of using NSAIDs, leading to greater patient understanding as compared to traditional drug information sources [97].

DIOAC‐GIB is notably different from the other risk scores, such as HAS‐BLED [12], ATRIA [20], HEMORR_2_HAGES [17], CBRM [109], and mOBRI [16]. First, most tools focus on any type of bleeding, not specific GIB. Although other types of bleeding are important, the gastrointestinal tract is one of the most common sites for bleeding. Second, nearly all the risk factors included in these models are not modifiable and therefore provide little guidance to clinicians when considering therapeutic options. Despite ample evidence that medications are associated with increased bleeding, none of the aforementioned tools incorporate specific chemical entities, and some do not include any medications. For example, the HAS‐BLED tool incorporates a single factor for medications but does not distinguish between two different types of medications, such as aspirin and NSAIDs [21]. In addition, the existing bleeding risk models have been developed using a single source of data and are therefore limited by the population studied and the variables included in the data [60, 82].

Some might question the usefulness of the DIOAC‐GIB model because clinicians counsel patients on OACs to avoid NSAIDs completely. However, this is not the reality of clinical practice. A cohort study conducted in South Korea found that 66% of 2283 patients who experienced major bleeding associated with direct OACs were exposed to known interacting medications, including NSAIDs (41%) and SSRIs (13%) [110]. A matched pair case‐control study of hospitalized Medicaid recipients in New York found that interactions involving warfarin with either NSAIDs or SSRIs were associated with over a 100% increase in the risk of hospitalization [111]. Another study that was a case analysis of over 1500 deaths attributable to adverse drug reactions (ADRs) found that the most common event was gastrointestinal hemorrhage [112]. Accounting for all types of ADR events, the authors concluded that reducing exposure to NSAIDs while taking an anticoagulant agent would be the most impactful way to reduce fatal ADRs. In another study using a cross‐sectional analysis of electronic health records reported a significant number of patients taking DOACs were co‐prescribed SSRIs/SNRIs (15%) and NSAIDs (10%) [113]. A retrospective cohort study found that nearly one‐third of patients were taking DOACs and aspirin concomitantly, and when compared with a matched cohort without aspirin, patients on both medications had a significant increase (*p* = 0.01) in the number of bleeding events, especially GIB, and an increased number of bleeding‐related hospitalizations [114]. In patients receiving DOACs, exposure to one interacting medication was associated with a 2‐fold (OR = 2.10 95% CI, 1.91–2.32) increased risk of major bleeding, while two or more interacting medications were associated with a 4‐fold (OR = 4.13, 95% CI, 3.72–4.59) increased risk of bleeding [110]. Specifically, patients taking DOACs and SSRIs have an increased bleeding risk that is nearly 2‐fold (OR = 1.91 95% CI, 1.29–2.83) higher than patients on a DOAC alone [7]. Our model was developed using data from multiple sources, including meta‐analyses, clinical trials, and observational studies, allowing us to synthesize the evidence across many settings to obtain a more generalizable risk estimate. One important consideration with this model is that it could help clinicians facilitate shared decision‐making with patients, visually demonstrating the risk of various therapeutic options and enhancing comprehension of the risk–benefit calculus with concomitant therapy.

## 5 Limitations

There are several limitations that should be considered regarding DIOAC‐GIB. The model was calibrated to predict GIB based on using the DOAC pivotal studies on AF. We restricted DOAC‐related GIB analyses to pivotal studies because these were large studies that systematically examined bleeding risk. These studies may not reflect bleeding risk in all types of patients receiving DOACs. The model has only been evaluated using a single data source; further studies should be undertaken and risk estimates refined. We also recognize that the model does not include all medications that interact with OACs, especially those with pharmacokinetic interactions [115]. Instead, we chose to focus on pharmacodynamic interactions because there is evidence for these products on the risk of bleeding and varying degrees of risk depending on the product selected. Another limitation is that there is considerable variation in the definition of a GIB in the literature. Some reports included ulcer and perforation [39], others used more broad terms such as: “gastro*intestinal bleeding from a duodenal or ulcer, acute lesions of the gastric mucosa, erosive duodenitis, or mixed lesions were considered for inclusion*” [61] or other definitions [37]. Many studies used diagnosis codes [116]. Studies have validated these GIB diagnosis codes to minimize bias [117, 118]. Only those studies that reported GIB were included in our evidence evaluation. Another important consideration is that the model does not specify the risk associated with individual P2Y12 products because the evidence for these products was based on aspirin as the control, limiting the ability to estimate the independent risk of each product. Also, the model does not account for time of exposure. Although it is common to express risk based on person‐years or a similar metric, the large body of evidence that was used to develop the model lacked data on time associated with the risk estimate. Finally, our risk model incorporates some but not all possible risk factors for GIB, such as uncontrolled hypertension. A recent analysis of the various risk factors for GIB with OACs highlights the potential for additional characteristics to consider in future iterations of the model [119]. Our model included age and a history of a previous GIB but did not include gender because the published evidence is limited and conflicting. We also did not include alcohol use because this was not perceived to be routinely captured in EHRs at the time the experts identified the risk factors [120]. Other risk models like HAS‐BLED include factors such as uncontrolled hypertension and other comorbidities that are largely nonmodifiable. Incorporation of these characteristics may be beneficial for clinicians and patients to consider when evaluating the risk of GIB.

## 6 Conclusion

DIOAC‐GIB is a unique novel risk model that allows clinicians to visualize the impact on the risk of GIB when modifying the patient's medications, considering patient‐specific characteristics. Unlike previous models of bleeding, DIOAC‐GIB incorporates the risk of GIB for each specific NSAID and antidepressant as well as other antiplatelet products and oral corticosteroids to allow clinicians and patients to evaluate alternatives and reduce harm due to OAC DDI based on patient‐specific characteristics and co‐medications. DIOAC‐GIB is a significant change in the approach to assessing and managing DDIs and helps to quantify and visualize GIB risk as compared to current DDI warnings that use terms such as “*major*”, “*serious*”, or “*contraindicated*”. Furthermore, the model corresponds well to HAS‐BLED and DOAC with respect to classification of patients at high risk of bleeding.

## Author Contributions

**Ainhoa Gomez‐Lumbreras:** investigation, writing – original draft, methodology, writing – review and editing, formal analysis, data curation. **Abdelrahman G. Tawfik:** investigation, methodology, writing – review and editing, visualization, validation, software, formal analysis, data curation. **Thomas Reese:** investigation, funding acquisition, methodology, writing – review and editing. **Daniel M. Witt:** investigation, writing – review and editing, resources. **Guilherme Del Fiol:** investigation, funding acquisition, writing – review and editing, methodology. **Daniel C. Malone:** conceptualization, investigation, funding acquisition, writing – original draft, methodology, visualization, writing – review and editing, validation, project administration, supervision, resources.

## Funding

This project has been supported in part by the Agency for Health Care Research and Quality (AHRQ) (Award Number R18HS029300, Implementation of DDInteract: A Shared‐decision Making Tool for Anticoagulant Drug–Drug INTERACTions). The content is solely the responsibility of the authors and does not represent the official views of the AHRQ.

## Disclosure

AI Use Statement: The authors did not use generative artificial intelligence or AI‐assisted technologies in the preparation of this manuscript.

## Conflicts of Interest

The authors declare no conflicts of interest with respect to the research, authorship, and/or publication of this article.

## Supporting information


**Table S1:** Search terms for literature used for metaanalyses.
**Table S2:** Characteristics of the included studies.
**Figure S1:** (a) Funnel plot for History of Gastrointestinal Bleeding. (b) Funnel plot of Gastrointestinal Bleeding for Antiplatelet Users. (c) Funnel plot of Gastrointestinal Bleeding for Corticosteroid Users.
**Figure S2:** Display of DDInteract as Implemented in an Electronic Health Record System.
**Figure S3:** Relationship between HAS‐BLED Score and DIOAC‐GIB Predicted Risks.
**Figure S4:** Relationship between DOAC Score and DIOAC‐GIB Predicted Risks.

## Data Availability

The data that support the findings of this study are available from the corresponding author upon reasonable request.
