# Supplementary material for: A Model for Estimating the Risk of Gastrointestinal Bleeding for Patients on Oral Anticoagulants and Interacting Medications
Source: Pharmacotherapy. 2026 Oct 25;46(11):e70205. doi: 10.1002/phar.70205 (PMC13696013; doi:10.1002/phar.70205)
Supplement: Supplementary file 1 [file PHAR-46-0-s001.docx]

**Table S1. Search terms**

| **Factor** | **Search Strategy** |
| --- | --- |
| Age | ((age) AND (gastrointestinal bleed*)) OR ((age) AND (gastrointestinal hemorrhage [MeSH Terms]))" |
| Antiplatelets | ((antiplatelets) AND (gastrointestinal bleed*)) OR ((platelet aggregation inhibitors) AND (gastrointestinal hemorrhage [MeSH Terms])) |
| clopidogrel | ((clopidogrel) AND (gastrointestinal bleed*)) OR ((clopidogrel) AND (gastrointestinal hemorrhage [MeSH Terms])) |
| prasugrel | ((prasugrel) AND (gastrointestinal bleed*)) OR ((prasugrel hydrochloride) AND (gastrointestinal hemorrhage [MeSH Terms])) |
| ticagrelor | ((ticagrelor) AND (gastrointestinal bleed*)) OR ((ticagrelor) AND (gastrointestinal hemorrhage [MeSH Terms])) |
| cangrelor | ((cangrelor) AND (gastrointestinal bleed*)) OR ((cangrelor) AND (gastrointestinal hemorrhage [MeSH Terms])) |
| Corticosteroids | ((corticosteroids) AND (gastrointestinal bleed*)) OR ((glucocorticoids) AND (gastrointestinal hemorrhage [MeSH Terms])) |
| prednisone | ((prednisone) AND (gastrointestinal bleed*)) OR ((prednisone) AND (gastrointestinal hemorrhage [MeSH Terms])) |
| prednisolone | ((prednisolone) AND (gastrointestinal bleed*)) OR ((prednisolone) AND (gastrointestinal hemorrhage [MeSH Terms])) |
| methylprednisolone | ((methylprednisolone) AND (gastrointestinal bleed*)) OR ((methylprednisolone) AND (gastrointestinal hemorrhage [MeSH Terms])) |
| dexamethasone | ((dexamethasone) AND (gastrointestinal bleed*)) OR ((dexamethasone) AND (gastrointestinal hemorrhage [MeSH Terms])) |
| deflazacort | ((deflazacort) AND (gastrointestinal bleed*)) OR ((deflazacort) AND (gastrointestinal hemorrhage [MeSH Terms])) |
| hydrocortisone | ((hydrocortisone) AND (gastrointestinal bleed*)) OR ((hydrocortisone) AND (gastrointestinal hemorrhage [MeSH Terms])) |
| PPI | ((PPI) AND (gastrointestinal bleed*)) OR (proton pump inhibitors) AND (gastrointestinal bleed*)) OR ((proton pump inhibitors) AND (gastrointestinal hemorrhage [MeSH Terms])) |
| omeprazole | ((omeprazole) AND (gastrointestinal bleed*)) OR ((omeprazole) AND (gastrointestinal hemorrhage [MeSH Terms])) |
| esomeprazole | ((esomeprazole) AND (gastrointestinal bleed*)) OR ((esomeprazole) AND (gastrointestinal hemorrhage [MeSH Terms])) |
| pantoprazole | ((pantoprazole) AND (gastrointestinal bleed*)) OR ((pantoprazole) AND (gastrointestinal hemorrhage [MeSH Terms])) |
| rabeprazole | ((rabeprazole) AND (gastrointestinal bleed*)) OR ((rabeprazole) AND (gastrointestinal hemorrhage [MeSH Terms])) |
| lansoprazole | ((lansoprazole) AND (gastrointestinal bleed*)) OR ((lansoprazole) AND (gastrointestinal hemorrhage [MeSH Terms])) |
| History of Gastrointestinal Bleeding | ((history of gastrointestinal bleed*) AND (gastrointestinal bleed*)) OR ((age) AND (gastrointestinal hemorrhage [MeSH Terms])) |

**Evidence supporting the estimated risk of Gastrointestinal bleeding for each of the selected risk factors**

**Table S2.** Characteristics of the included studies

| Study | Exposure | Design | Setting | Time Period | Risk estimated | GIB definition | Sample size | Sex Male (%) | Age (years) |
| --- | --- | --- | --- | --- | --- | --- | --- | --- | --- |
| Agudo-Fernández 2021^1^ | Antiplatelets  Corticosteroids  History of GIB | Cohort: RHEDAR study | Rey Juan Carlos Hospital.  Madrid, Spain | 2012-2016 | OR (95%CI) | Gastrointestinal bleeding events (melena, hematemesis, rectal bleeding, or hematochezia) | 1,213 | 52.7 | 72.6 ± 14.6 |
| Ahn 2022^2^ | PPI | Meta-analysis | Ten studies | Inception to 2022 | OR (95%CI) | Incidence of total GIB: total GIB | 1,970,931 | N/A | N/A |
| Alexander 1985^3^ | Corticosteroids | Cohort | Hereford County Hospital, United Kingdom | 1977-1980 | OR | ICD-8 codes (gastro-intestinal bleeding and/or ulceration) | 1,878 | N/A | >75 |
| Blot 2000^4^ | History of GIB | Case control | American College of Gastroenterology | 1995 | OR  (95% CI) | Survey and gastroenterologist review | 1,217 | Cases 62.7; Controls 49.3 | +>65 Cases 44.8; Controls 33.2 |
| Carvajal 2011^5^ | History of GIB | Case control | Spain (four hospitals) and Italy (one hospital) | 2004-2007 | OR  (95% CI) | Endoscopy acute UGIB from a duodenal or gastric ulcer, acute lesions of the gastric mucosa, erosive duodenitis, or mixed lesions | 1,939 | 71.3 | Mean 62.6 (SD 17.0) |
| Chan 2015^6^ | Antiplatelets | Cohort | Clinical Data Analysis and Reporting System (CDARS) of the Hong Kong Hospital Authority (HA) | 2010-2013 | IRR  (95% CI) | ICD -9 Clinical Modification | 5,041 | 47.8 | 72.0 ± 10.9 |
| Chen 2014^7^ | Age  History of GIB | Cohort | Kaohsiung Veterans General Hospital. Kaohsiung City, Taiwan | 1993-2012 | RR  (95% CI) | Clinically evident hematemesis, melena, and hematochezia, or positive for occult blood test | 401 | 58.3 | 65.2±16.6 |
| Cheng 2015^8^ | Corticosteroids  Antiplatelets | Cohort | National Health Insurance Research Database (NHIRD).  Taiwan | 2000 - 2010 | Hazard Ratio  (95% CI) | ICD-9-CM codes for UGIB ad LGIB | 48,765 | Controls 38.36; SSRI 38.35; and SNRI 8.45 | Controls 50.36±17.33; SSRI 50.44±17.44 and SNRI 49.85±16.51 |
| Connolly 2009^9^ | Dabigatran | RCT (RELY) | Worldwide (44 countries) | 2005 - 2007 | Rate of GIB | Major GIB (GIB could be life threatening or non-life threatening) | 18,113 | 63.2 | 71.5±8.8 |
| Crooks 2013^10^ | Corticosteroids  Antiplatelets | Case control | GPRD, UK | 1997-2010 | OR  (95% CI) | Defining upper gastrointestinal bleeding  from linked primary and secondary care data and the effect on occurrence  and 28-day mortality | 97,824 | NA | NA |
| Dall 2009^11^ | Antiplatelets  Corticosteroids  Hx of GIB | Case crossover | Denmark. Funen County Patient Administrative System (FPAS), Odense University Pharmaco-epidemiological Database (OPED), Danish Central Person Register (CPR). | 1995 - 2006 | OR  (95% CI) | Peptic ulcer or gastritis + Significant bleeding defined by either melena, a subnormal hemoglobin, or the need for transfusions + bleeding source in the stomach or duodenum identified by endoscopy or surgery | 40,154 | Cases 51.2; | 72.1 ± 14.1 |
| De Abajo 2013^12^ | Antiplatelets  Corticosteroids  History of GIB | Case control | BIFAP (Primary care database), Spain | 2001 - 2005 | Rate ratio (95% CI) | International Classification of Primary Care (ICPC) codes or a free-text related to upper gastrointestinal bleeding | 11,193 | Cases 63.8 and controls 64.4 | Cases 65.0 (±13.2) and controls 64.3 (±13.2) |
| Delaney 2007^13^ | Antiplatelets | Case-control | General Practice Research Database (GPRD)  United Kingdom | 2000- 2005 | Rate ratio (95% CI) | Computerized code for gastrointestinal bleeding (codes not provided) | 44,199 | Cases 53.9 and controls 42.9 | Cases 69.3 (17.6) and controls 69.1 (17.7) |
| Garcia DA 2020^14^ | History of GIB | Post hoc analysis Aristotle RCT | Worldwide (39 countries) | 2006 - 2010 | HR  (95% CI) | GIB events (upper or lower) extracted from the case report form. | 18,201 | NA | NA |
| Garcia Rodriguez 1994^15^ | Corticosteroids  History of GIB | Case control | Value  Added Medical Products (VAMP) Research (London, UK). | 1990-1993 | Relative risk  (95% CI) | OXMIS code for UGIB and manually review | 11,457 | NA | NA |
| Garcia Rodriguez 1998^16^ | Corticosteroids  Antiplatelets | Case control | Automated Health care data files from the region of Friuli-Venezia Giulia. Italy | 1991 - 1995 | Relative risk  (95% CI) | UGIB hospitalization (primary discharge diagnosis of bleeding or perforated peptic ulcer) Diagnosis codes ICD-9 | 21,505 | Cases 63.2; Controls 46.9 | Cases >60 62.3; Controls >60 34.5 |
| Ghule 2024^17^ | Aspirin | Meta-analysis | No language restriction was  used. | inception to January 2021 | OR  (95% CI) | Gastrointestinal bleeding, gastrointestinal hemorrhage, GI | Subgroup Analysis of GI Bleeding of Randomized Controlled Trial for Oral  Anticoagulants Combined with Aspirin vs Oral Anticoagulants Alone | | |
| Giugliano 2013^18^ | Edoxaban | RCT (ENGAGE) | Worldwide (46 countries) | 2008 - 2010 | Rate of GIB | Gastrointestinal bleeding (upper and lower) | 21,105 | W 62.5; Edoxaban H 62.1; Edoxaban L 61.2; | 72 years |
| Gomez-Lumbreras 2025^19^ | Antidepressants | Meta-analysis |  | Inception to October 2025 | OR  (95% CI) | 20 studies | NA | NA | NA |
| Granger 2011^20^ | Apixaban | RCT (ARISTOTLE) | Worldwide (39 countries) | 2006 - 2010 | Rate of GIB | Major Gastrointestinal Bleeding (ISTH) | 18,201 | A 64.5; W 65 | 70  (IQR 63–76) |
| Grymonprez 2025^21^ | Corticosteroids  Antiplatelets | Cohort | InterMutualistic Agency  (IMA) database and Minimal Hospital Dataset (MHD). Belgium | 2013 - 2019 | HR  (95% CI) | ICD-9 and ICD- 10 codes | 79,760 | No DDI 50.9 and DDI 51 | No DDI 75.6 ± 10.1; DDI 75.9 ± 10.9 |
| Guo 2019^22^ | Corticosteroids  Antiplatelets | Cohort | Clinical Data Analysis and Reporting System (CDARS) of the Hong Kong Hospital Authority. Hong Kong | 2003-2012 | HR  (95% CI) | hospitalization for nonvariceal  UGIB by ICD‐9 codes | 10,074 | SSRI 30.9; non-SSRI 31.2 | SSRI 53 (45‐65); non-SSRI 54 (45‐66) |
| Gutthann 1997^23^ | History of GIB | Cohort | Saskatchewan Hospital Services Plan (Canada) | 1982-1986 | OR  (95% CI) | Chart review of computerized records | 11,377 | Cases 47.8% and controls 43.4 | < 60 Cases 22.3% and controls 56.2% |
| Hallas 2006^24^ | Antiplatelets  History of GIB | Case-control | Databases:  - Funen County Patient Administrative System (FPAS)  - Odense University Pharmaco-epidemiological Database (OPED)  - Danish Central Person Register (CPR)  Denmark | 2000-2004 | OR  (95% CI) | ICD-10 codes + manually reviewed discharge summaries | 59,163 | Cases and controls 49.2 | Cases 72.4 (14.3) and controls 71.8 (14.3) |
| Hansen 2010^25^ | Antiplatelets | Cohort | Nationwide administrative registries:  - Danish National Registry of Patients  - Register of Medicinal Product Statistics  - Danish Civil Registration System  Denmark | 1997-2006 | HR  (95% CI) | ICD-10 codes: gastrointestinal bleedings K25.0, K25.4, K26.0, K26.4, K27.0, K28.0, K92.0, K92.1, and K92.2) | 126,837 | Warfarin plus clopidogrel (69.9) and clopidogrel (60.0) | Warfarin plus clopidogrel (70.2 (8.9) and clopidogrel (72.6 (10.2) |
| Hochain 1995^26^ | History of GIB  Corticosteroids | Case-control | Gastroenterology Unit. Hospital Center University De Rouen.  Rouen, France | 1992-1998 | OR (95%CI) | Hospital admission for upper GIB confirmed by endoscopy | 360 | NA | NA |
| Holt 2022^27^ | Corticosteroids | Case-control | Nationwide registries:   - Danish National Patient Register - National Causes of Death Register - National Prescription Register   Denmark | 2012–2018 | HR (95% CI) | ICD-10 codes | 98,376 | 56.1 | 75 (68 – 82) |
| Hreinsson 2013^28^ | Corticosteroids  Antiplatelets | Cohort | National University Hospital of Iceland | 2009-2010 | OR manually estimated from reported data | Upper gastrointestinal endoscopy and/or colonoscopy | 132 | 55.3 | NA |
| Ibáñez 2006^29^ | Antiplatelets  History of GIB | Case-control | Spain (10 hospitals) and Italy (8 hospitals) | Spain 1998 – 2001; Italy 1999 – 2001 | OR (95% CI) | Review of all records of all endoscopic procedures. | 10,006 | NA | NA |
| Kurlander 2022^30^ | PPI | Meta-analysis | Seven studies | Inception to April 7, 2021 | RR  (95% CI) | Gastrointestinal hemorrhage | NA | NA | NA |
| Lanas 2003^31^ | History of GIB | Case control | Four general hospitals in Spain | 1995-1998 | OR manually estimated from reported data | Patients admitted with bleeding from a peptic lesion (including ulcers, erosions and acute mucosal lesions). | 3,353 | Cases 69.3 and controls 69.2 | Cases 65.1 (16.6) controls 65.2 (16.7) |
| Lanas 2006^32^ | History of GIB  Antiplatelets | Case control | Network of general hospitals integrated within the Spanish Association of Gastroenterology. Spain | 2001-2004 | Relative risk | Patient hospitalised because of gastrointestinal bleeding (haematemesis or melena), which was confirmed by hospital personnel through an endoscopic diagnosis as caused by peptic ulcer lesion. | 8,309 | NA | 61 |
| Lanas 2015^33^ | Antiplatelets | Case control | General hospitals integrated  within the Spanish Association of Gastroenterology and  the Biomedical Investigation Network Center of hepatic  and digestive diseases (CIBERehd). | 2009 - 2013 | Crude RR  (95% CI) | Hematemesis, melena, hematochezia, or red blood per rectum), which was confirmed by hospital personnel | 2016 | 60.7 | >50 years 83.2 |
| Laporte 1991^34^ | History of GIB | Case control | Hospital  Vall d’Hebron, Barcelona; Hospital de Sant Pau, Barcelona; and  Hospital Son Dureta, Palma de Mallorca  Spain | 1987-1988 | OR  (95% CI) | hematemesis or melena and a primary diagnosis of acute upper gastrointestinal bleeding  Endoscopy plus survey | 3,557 | 72% | >60 years = 25.1% |
| Laporte 2004^35^ | Corticosteroids  Antiplatelets | Case controls | Spain (10 hospitals) Italy (8 hospitals) | 1998 – 2001 | OR  (95% CI) | Records of all endoscopic procedures and lists of admission diagnosis were examined daily. | 10,006 | NA | >18 |
| Li 2024^36^ | Antiplatelets | Case-control | Taiwan National health Insurance Program | 2000 – 2013 | OR  (95% CI) | hospitalization for UGIB; (ICD-9-CM | 2,512 | Cases 54; control 54 | Cases 79.0 (6.0); controls 78.8 (6.0) |
| Lin 2011^37^ | Antiplatelets  Corticosteroids  History of GIB | Case-control | The Health Improvement Network (THIN) database in the United Kingdom | 2000-2007 | RR  (95% CI) | Computerized patient profiles of all potential cases (including free-text comments) manually reviewed | 22,049 | NA | 40–84 |
| Lin 2013^38^ | Antiplatelets | Cohort | Taiwan National Health Insurance Research Database (NHIRD) | 1996-2010 | HR  (95% CI) | ICD-9 codes for nonvariceal UGIB | 16,190 | Controls 58.65 and clopidogrel 58.65 | Controls 68.56 (11.62) and clopidogrel 68.56 (11.62) |
| Luo 2019^39^ | Corticosteroids | Cohort | National Health Insurance Research Database (NHIRD). Taiwan | 1996-2010 | HR  (95% CI) | ICD-9 codes | 22.210 | Controls 25.2 and aspirin 52.81 | 64.26 (12.63) and aspirin 64.03 (12.67) |
| Masclee 2014^40^ | Corticosteroids  Antiplatelets | Meta-analysis | Databases:   - Integrated Primary Care Information (IPCI). The Netherlands, - Health Search/CSD Longitudinal Patient Database (HSD). Italy - Pedianet. Italy - Aarhus University Hospital Database. Denmark - PHARMO Institute. The Netherlands - Regional database of Lombardy (UNIMIB). Italy - Regional database of Tuscany (ARS). Italy.   Europe | NA | Incidence Rate Ratio (IRR) 95% CI | Pertinent disease  codes from the different coding systems in each database | 114,835 | NA | NA |
| Nantsupawat 2018^41^ | Corticosteroids  History of GIB | Cohort | University Medical Center, Texas Tech University Health Sciences Center.  Lubbock, Texas | 2010-2013 | OR (95%CI) | Hospitalized patients | 247 | 51.8 | 72 |
| Narum 2014^42^ | Corticosteroids | Meta-analysis | Worldwide (159 studies) | 1983 - 2011 | OR  (95% CI) | Complications of gastroduodenal ulcers. | 8, 651 | NA | NA |
| Nijenhuis 2020^43^ | Antiplatelets | RCT for transcatheter aortic valve implantation | The Netherlands, Belgium, Luxemburg, Czech Republic |  | OR manually estimated from reported data | Non–procedure-related bleeding (no specific definition): Bleeding events were classified according to the Valve Academic Research  Consortium-2 (VARC-2) | 313 | Placebo 56.1; clopidogrel 53.2 | Placebo 80.9±6.2; clopidogrel 81.0±5.5 |
| Nørgård 2004^44^ | Corticosteroids | Case control | Danish county of North Jutland | 2000 - 2002 | OR  (95% CI) | Upper gastrointestinal bleeding diagnostic  codes from ICD-10 | 3,686 | Cases 57.1; Controls 53.1 | Cases 66.8 (15.9); Controls 72.5 (13.5) |
| Opatrny 2008^45^ | Antiplatelets  Corticosteroids | Case-control | General Practice Research Database (GPRD) in the United Kingdom | 2000-2005 | Rate ratio (95% CI) | First diagnosis of upper GIB identified using a READ or OMXIS medical code | 44,199 | Cases 53.9; controls 42.9 | Cases 69.3/17.6; controls 69.1/17.7 |
| Otani 2022^46^ | History of GIB | Cohort | Osaka City University Hospital, Japan | 2004-2015 | HR  (95% CI) | Rebleeding after capsule endoscopy for obscure GIB described as bleeding with melena or hematochezia | 389 | 50.4 | 69.0 (59.0-76.0) |
| Patel 2011^47^ | Rivaroxaban | RCT (ROCKET AF) | Worldwide (45 countries) | 2006 - 2009 | Rate of GIB | Major bleeding from a gastrointestinal site (upper, lower and rectal) | 7,111 | 60.3 | 73 (65-78) |
| Reinau 2018^48^ | Corticosteroids  Antiplatelets | Case-control | Swiss health insurance company  Helsana | 2012-2016 | OR  (95% CI) | ICD-10 codes | 12,712 | Cases 52.6; controls 51.3 | Cases 74.1 (13.3); controls 74.1 (12.9) |
| Ruff 2014^49^ | Warfarin | Meta-analysis | Worldwide | 2009-2013 | RR  (95% CI) | NA | 71,683 | Warfarin 71.5 | Warfarin 73% |
| Schelleman 2011^50^ | History of GIB | Case-control | Medicaid  data of California, Florida, New York, Ohio, and Pennsylvania | 1999 - 2005 | OR (95%CI) | ICD-9 codes | 666,235 | Case 34.3; controls 36.5 | Cases ≥ 60 10,969; controls ≥ 60 487,887 |
| Sherid 2015^51^ | History of GIB  Antiplatelets | Cohort | CGH Medical Center. Sterling, Illinois  Saint Francis Hospital.  Evanston, Illinois | 2010-2012 | OR (95%CI) | GIB confirmed by endoscopy or other diagnostic methods | 417 | Dabigatran 50; Warfarin 45.9 | Dabigatran 72.72; Warfarin 71.83 |
| Sherwood 2015^52^ | History of GIB  Antiplatelets | Post hoc analysis Rocket AF | Worldwide (45 countries) | 2006 - 2009 | HR  (95% CI) | Same as the ROCKET AF trial | 14,236 | 60 | 73 (65 - 78) |
| Shimomura 2018^53^ | Antiplatelets | Cohort | National Center for Global  Health and Medicine, Tokyo, Japan, | 2001-2015 | HR  (95% CI) | GI-bleeding-related lesion by endoscopy | 508 | 64.8 | 69.4 (±9.9) |
| Targownik 2009^54^ | Antiplatelets  Corticosteroids | Case-control | Manitoba Population Health Research Data Repository.  Canada | 1995-2007 | OR  (95% CI) | Subjects who were admitted to the hospital with a most responsible diagnosis consistent with UGIB. | 70,142 | Cases 57.6 and controls 56.4 | Cases 68.9 ± 15.2 and controls 69.8 ± 14.7 |
| Tawfik 2025^55^ | NSAIDs | Meta-analysis |  | Inception to January  2025 | OR  (95% CI) | 25 studies | NA | NA | NA |
| Tseng 2015^56^ | Corticosteroids | Case-crossover study | National Health Insurance Research Database (NHIRD).  Taiwan | 2000-2012 | OR  (95% CI) | ICD-9 codes | 17,788 | Cases 64.5 | Cases 62.5 ± 17.4 |
| Van Walraven 2002^57^ | Corticosteroids  History of GIB | Cohort | Ontario, Canada. Ontario Drug Benefit Program, Administrative databases for physician and hospital services, Ontario Registered Persons Database | Surveillance starts 1992 | HR  (95% CI) | ICD-9 codes: Admissions to hospital with primary diagnosis of upper gastrointestinal bleeding identified from discharge abstract database. | 317,824 | NA | NA |
| Verdel 2011^58^ | Antiplatelets  Corticosteroids | Case-control | PHARMO RLS  Netherlands | 1998 - 2007 | OR  (95% CI) | ICD-9 codes | 24,844 | 18 | 52.3 |
| Vonbach 2007^59^ | Corticosteroids | Case-control | Kantonsspital Winterthur, Switzerland | 2005 | OR  (95% CI) | ICD-10 codes | 222 | 54 | >=70 68.9% |
| Wan Ghazali 2021^60^ | Antiplatelets  Corticosteroids | Cohort | Hospital Raja Perempuan Zainab II. Kota Bharu, Malasia | 2018 | HR  (95% CI) | Endoscopic confirmed GIB | 403 | 89.1 | 55.7 (16.3) |
| Weil 2000^61^ | Corticosteroids | Case-control | Hospitals in Glasgow, Newcastle, Nottingham, Oxford, Portsmouth.  United Kingdom | 1986-1991 | OR  (95% CI) | Hematemesis  or melena due to gastric or duodenal  ulceration | 2,110 | NA | NA |
| Wessinger 2006^62^ | Antiplatelets | Case-control | Northwestern Memorial Hospital and Rush  University Medical Center in Chicago, IL and Mayo  Clinic Hospital in Phoenix, AZ. USA | 2003-2004 | OR  (95% CI) | ICD-9 codes for gastrointestinal bleeding | 1,579 | Cases 49.6; Controls 49.7 | Cases 65.8 ±0.8; Controls 4.7 ± 0.5 |
| Yao 2020^63^ | Corticosteroids | Self-controlled case series | National Health Insurance Research Database (NHIRD).  Taiwan | 2013-2015 | IRR (95% CI) | ICD-9 codes | 2,623,327 | 44.7 | 38.0 (12.6) |

CI: confidence interval; DDI: drug-drug interactions; GIB: gastrointestinal bleeding; HR: hazard ratio; ICD: International Classification of Diseases; IRR: incidence rate ratio; ISTH: International Society on Thrombosis and Haemostasis; IRR: Incident rate ratio; NA : not applicable; NSAIDs: non-steroidal anti-inflammatory drugs; OR: odds ratio; PPI: proton pump inhibitors; RCT: randomized controlled trial; SD: standard deviation; SNRI: Serotonin–norepinephrine reuptake inhibitor; SSRI: Selective serotonin reuptake inhibitor.

**REFERENCES TABLE S2**

1.	Agudo‐Fernández S, Castaño Milla C, González Blanco A, Olmos Jerez JA, Calvo Morillas I, Sancho Del Val L. RHEDAR study: Determination of the risk of gastrointestinal hemorrhage in treatment with dabigatran, acenocoumarol and rivaroxaban. *J Gastroenterol Hepatol*. 2021;36(10):2794-2802. doi:10.1111/jgh.15547

2.	Ahn H, Lee S, Choi E, et al. Protective effect of proton‐pump inhibitor against gastrointestinal bleeding in patients receiving oral anticoagulants: A systematic review and meta‐analysis. *Br J Clin Pharmacol*. 2022;88(11):4676-4687. doi:10.1111/bcp.15478

3.	Alexander AM, Veitch GBA, Wood JB. Anti-rheumatic and analgesic drug usage and acute gastro-intestinal bleeding in elderly patients. *J Clin Hosp Pharm*. 1985;10(1):89-93.

4.	Blot WJ, McLaughlin JK. Over the counter non-steroidal anti-inflammatory drugs and risk of gastrointestinal bleeding. *J Epidemiol Biostat*. 2000;5(2):137-142.

5.	Carvajal A, Ortega S, Del Olmo L, et al. Selective Serotonin Reuptake Inhibitors and Gastrointestinal Bleeding: A Case-Control Study. Wright JM, ed. *PLoS ONE*. 2011;6(5):e19819. doi:10.1371/journal.pone.0019819

6.	Chan EW, Lau WCY, Leung WK, et al. Prevention of Dabigatran-Related Gastrointestinal Bleeding With Gastroprotective Agents: A Population-Based Study. *Gastroenterology*. 2015;149(3):586-595.e3. doi:10.1053/j.gastro.2015.05.002

7.	Chen W chi, Chen Y hua, Hsu P i, et al. Gastrointestinal Hemorrhage in Warfarin Anticoagulated Patients: Incidence, Risk Factor, Management, and Outcome. *Biomed Res Int*. 2014;2014(463767). doi:10.1155/2014/463767

8.	Cheng YL, Hu HY, Lin XH, et al. Use of SSRI, But Not SNRI, Increased Upper and Lower Gastrointestinal Bleeding: A Nationwide Population-Based Cohort Study in Taiwan. *Medicine (Baltimore)*. 2015;94(46):e2022. doi:10.1097/MD.0000000000002022

9.	Connolly SJ, Ezekowitz MD, Yusuf S, et al. Dabigatran versus Warfarin in Patients with Atrial Fibrillation. *N Engl J Med*. 2009;361(12):1139-1151. doi:10.1056/NEJMoa0905561

10.	Crooks CJ, West J, Card TR. Comorbidities Affect Risk of Nonvariceal Upper Gastrointestinal Bleeding. *Gastroenterology*. 2013;144(7):1384-1393.e2. doi:10.1053/j.gastro.2013.02.040

11.	Dall M, Schaffalitzky De Muckadell OB, Lassen AT, Hansen JM, Hallas J. An Association Between Selective Serotonin Reuptake Inhibitor Use and Serious Upper Gastrointestinal Bleeding. *Clin Gastroenterol Hepatol*. 2009;7(12):1314-1321. doi:10.1016/j.cgh.2009.08.019

12.	De Abajo FJ, Gil MJ, Bryant V, Timoner J, Oliva B, García-Rodríguez LA. Upper gastrointestinal bleeding associated with NSAIDs, other drugs and interactions: a nested case–control study in a new general practice database. *Eur J Clin Pharmacol*. 2013;69(3):691-701. doi:10.1007/s00228-012-1386-3

13.	Delaney JA, Opatrny L, Brophy JM, Suissa S. Drug drug interactions between antithrombotic medications and the risk of gastrointestinal bleeding. *Can Med Assoc J*. 2007;177(4):347-351. doi:10.1503/cmaj.070186

14.	Garcia DA, Fisher DA, Mulder H, et al. Gastrointestinal bleeding in patients with atrial fibrillation treated with Apixaban or warfarin: Insights from the Apixaban for Reduction in Stroke and Other Thromboembolic Events in Atrial Fibrillation (ARISTOTLE) trial. *Am Heart J*. 2020;221:1-8. doi:10.1016/j.ahj.2019.10.013

15.	García Rodríguez L, Jick H. Risk of upper gastrointestinal bleeding and perforation associated with Individual non-steroidal anti-inflammatory drugs. *The Lancet*. 1994;343(8900):769-772. doi:10.1016/S0140-6736(94)91843-0

16.	García Rodríguez LA, Cattaruzzi C, Troncon MG, Agostinis L. Risk of Hospitalization for Upper Gastrointestinal Tract Bleeding Associated With Ketorolac, Other Nonsteroidal Anti-inflammatory Drugs, Calcium Antagonists, and Other Antihypertensive Drugs. *Arch Intern Med*. 1998;158(1):33. doi:10.1001/archinte.158.1.33

17.	Ghule P, Panic J, Malone DC. Risk of bleeding with concomitant use of oral anticoagulants and aspirin: A systematic review and meta-analysis. *Am J Health Syst Pharm*. Published online January 23, 2024:zxae010. doi:10.1093/ajhp/zxae010

18.	Giugliano RP, Ruff CT, Braunwald E, et al. Edoxaban versus Warfarin in Patients with Atrial Fibrillation. *N Engl J Med*. 2013;369(22):2093-2104. doi:10.1056/NEJMoa1310907

19.	Gomez‐Lumbreras A, Tawfik AG, Del Fiol G, et al. Risk of gastrointestinal bleeding by specific SSRIs and SNRIs: A systematic review and meta‐analysis. *Br J Clin Pharmacol*. 2026;92(3):793-808. doi:10.1002/bcp.70432

20.	Granger CB, Alexander JH, McMurray JJV, et al. Apixaban versus warfarin in patients with atrial fibrillation. *N Engl J Med*. 2011;365(11):981-992. doi:10.1056/NEJMoa1107039

21.	Grymonprez M, Capiau A, Steurbaut S, et al. Pharmacodynamic Drug-Drug Interactions and Bleeding Outcomes in Patients with Atrial Fibrillation Using Non-Vitamin K Antagonist Oral Anticoagulants: a Nationwide Cohort Study. *Cardiovasc Drugs Ther*. 2025;39(1):133-143. doi:10.1007/s10557-023-07521-5

22.	Guo CG, Chen L, Chan EW, et al. Systematic review with meta-analysis: the risk of gastrointestinal bleeding in patients taking third-generation P2Y12 inhibitors compared with clopidogrel. *Aliment Pharmacol Ther*. 2019;49(1):7-19. doi:10.1111/apt.15059

23.	Gutthann SP, GarcíaRodríguez LA, Raiford DS. Individual Nonsteroidal Antiinflammatory Drugs and Other Risk Factors for Upper Gastrointestinal Bleeding and Perforation: *Epidemiology*. 1997;8(1):18-24. doi:10.1097/00001648-199701000-00003

24.	Hallas J, Dall M, Andries A, et al. Use of single and combined antithrombotic therapy and risk of serious upper gastrointestinal bleeding: population based case-control study. *BMJ*. 2006;333(7571):726. doi:10.1136/bmj.38947.697558.AE

25.	Hansen ML, Sørensen R, Clausen MT, et al. Risk of Bleeding With Single, Dual, or Triple Therapy With Warfarin, Aspirin, and Clopidogrel in Patients With Atrial Fibrillation. *Arch Intern Med*. 2010;170(16):1433-1441. doi:10.1001/archinternmed.2010.271

26.	Hochain P, Berkelmans I, Czernichow P, et al. Which patients taking non-aspirin non-steroidal anti-inflammatory drugs bleed? A case-control study. *Eur J Gastroenterol Hepatol*. 1995;7(5):419-426.

27.	Holt A, Blanche P, Zareini B, et al. Gastrointestinal bleeding risk following concomitant treatment with oral glucocorticoids in patients on non-vitamin K oral anticoagulants. *Heart*. 2022;108(8):626-632. doi:10.1136/heartjnl-2021-319503

28.	Hreinsson JP, Kalaitzakis E, Gudmundsson S, Björnsson ES. Upper gastrointestinal bleeding: incidence, etiology and outcomes in a population-based setting. *Scand J Gastroenterol*. 2013;48(4):439-447. doi:10.3109/00365521.2012.763174

29.	Ibáñez L, Vidal X, Vendrell L, Moretti U, Laporte JR, THe Spanish-Italian collaborative group for the epidemiology of gastrointestinal bleeding. Upper gastrointestinal bleeding associated with antiplatelet drugs. *Aliment Pharmacol Ther*. 2006;23(2):235-242. doi:10.1111/j.1365-2036.2006.02759.x

30.	Kurlander JE, Barnes GD, Fisher A, et al. Association of Antisecretory Drugs with Upper Gastrointestinal Bleeding in Patients Using Oral Anticoagulants: A Systematic Review and Meta-Analysis. *Am J Med*. 2022;135(10):1231-1243.e8. doi:10.1016/j.amjmed.2022.05.031

31.	Lanas A, Serrano P, Bajador E, Fuentes J, Sáinz R. Risk of upper gastrointestinal bleeding associated with non-aspirin cardiovascular drugs, analgesics and nonsteroidal anti-inflammatory drugs: *Eur J Gastroenterol Hepatol*. 2003;15(2):173-178. doi:10.1097/00042737-200302000-00011

32.	Lanas A, García-Rodríguez LA, Arroyo MT, et al. Risk of upper gastrointestinal ulcer bleeding associated with selective cyclo-oxygenase-2 inhibitors, traditional non-aspirin non-steroidal anti-inflammatory drugs, aspirin and combinations. *Gut*. 2006;55(12):1731-1738. doi:10.1136/gut.2005.080754

33.	Lanas Á, Carrera-Lasfuentes P, Arguedas Y, et al. Risk of Upper and Lower Gastrointestinal Bleeding in Patients Taking Nonsteroidal Anti-inflammatory Drugs, Antiplatelet Agents, or Anticoagulants. *Clin Gastroenterol Hepatol*. 2015;13(5):906-912.e2. doi:10.1016/j.cgh.2014.11.007

34.	Laporte JR, Carné X, Vidal X, Moreno V, Juan J. Upper gastrointestinal bleeding in relation to previous use of analgesics and non-steroidal anti-inflammatory drugs. *The Lancet*. 1991;337(8733):85-89. doi:10.1016/0140-6736(91)90744-A

35.	Laporte JR, Ibanez L, Vidal X, Vendrell L, Leone R. Upper Gastrointestinal Bleeding Associated with the Use of NSAIDs: Newer Versus Older Agents. *Drug Saf*. 2004;27(6):411-420. doi:10.2165/00002018-200427060-00005

36.	Li YH, Hang LW, Muo CH, Chen SJ, Chen PC. Dose of selective serotonin reuptake inhibitors and risk of upper gastrointestinal bleeding in older adults. *J Psychopharmacol (Oxf)*. 2024;38(2):137-144. doi:10.1177/02698811231218955

37.	Lin KJ, Hernández–Díaz S, García Rodríguez LA. Acid Suppressants Reduce Risk of Gastrointestinal Bleeding in Patients on Antithrombotic or Anti-Inflammatory Therapy. *Gastroenterology*. 2011;141(1):71-79. doi:10.1053/j.gastro.2011.03.049

38.	Lin CC, Hu HY, Luo JC, et al. Risk factors of gastrointestinal bleeding in clopidogrel users: A nationwide population-based study. *Aliment Pharmacol Ther*. 2013;38(9):1119-1128. doi:10.1111/apt.12483

39.	Luo PJ, Lin XH, Lin CC, et al. Risk factors for upper gastrointestinal bleeding among aspirin users: An old issue with new findings from a population-based cohort study. *J Formos Med Assoc*. 2019;118(5):939-944. doi:10.1016/j.jfma.2018.10.007

40.	Masclee GMC, Sturkenboom MCJM, Kuipers EJ. A benefit-risk assessment of the use of proton pump inhibitors in the elderly. *Drugs Aging*. 2014;31(4):263-282.

41.	Nantsupawat T, Soontrapa S, Nantsupawat N, et al. Risk factors and prevention of dabigatran‐related gastrointestinal bleeding in patients with atrial fibrillation. *J Arrhythmia*. 2018;34(1):30-35. doi:10.1002/joa3.12015

42.	Narum S, Westergren T, Klemp M. Corticosteroids and risk of gastrointestinal bleeding: A systematic review and meta-analysis. *BMJ Open*. 2014;4(5):1-9. doi:10.1136/bmjopen-2013-004587

43.	Nijenhuis VJ, Brouwer J, Delewi R, et al. Anticoagulation with or without Clopidogrel after Transcatheter Aortic-Valve Implantation. *N Engl J Med*. 2020;382(18):1696-1707. doi:10.1056/NEJMoa1915152

44.	Nørgård B, Pedersen L, Johnsen SP, et al. COX‐2‐selective inhibitors and the risk of upper gastrointestinal bleeding in high‐risk patients with previous gastrointestinal diseases: a population‐based case–control study. *Aliment Pharmacol Ther*. 2004;19(7):817-825. doi:10.1111/j.1365-2036.2004.01913.x

45.	Opatrny L, Delaney JA ‘Chris’, Suissa S. Gastro‐intestinal haemorrhage risks of selective serotonin receptor antagonist therapy: a new look. *Br J Clin Pharmacol*. 2008;66(1):76-81. doi:10.1111/j.1365-2125.2008.03154.x

46.	Otani K, Shimada S, Watanabe T, et al. Long-term rebleeding rate and predictive factors of rebleeding after capsule endoscopy in patients with obscure GI bleeding. *Gastrointest Endosc*. 2022;96(6):956-969.e3. doi:10.1016/j.gie.2022.07.012

47.	Patel MR, Mahaffey KW, Garg J, et al. Rivaroxaban versus Warfarin in Nonvalvular Atrial Fibrillation. *N Engl J Med*. 2011;365(10):883-891. doi:10.1056/NEJMoa1009638

48.	Reinau D, Schwenkglenks M, Früh M, Signorell A, Blozik E, Meier CR. Glucocorticoids and the Risk of Peptic Ulcer Bleeding: Case–Control Analysis Based on Swiss Claims Data. *Drug Saf*. 2018;41(7):725-730. doi:10.1007/s40264-018-0645-3

49.	Ruff CT, Giugliano RP, Braunwald E, et al. Comparison of the efficacy and safety of new oral anticoagulants with warfarin in patients with atrial fibrillation: a meta-analysis of randomised trials. *Lancet Lond Engl*. 2014;383(9921):955-962. doi:10.1016/S0140-6736(13)62343-0

50.	Schelleman H, Brensinger CM, Bilker WB, Hennessy S. Antidepressant-Warfarin Interaction and Associated Gastrointestinal Bleeding Risk in a Case-Control Study. Laks J, ed. *PLoS ONE*. 2011;6(6):e21447. doi:10.1371/journal.pone.0021447

51.	Sherid M, Sifuentes H, Sulaiman S, et al. Gastrointestinal Bleeding with Dabigatran, a Comparative Study with Warfarin: A Multicenter Experience. *Korean J Gastroenterol*. 2015;65(4):205. doi:10.4166/kjg.2015.65.4.205

52.	Sherwood MW, Nessel CC, Hellkamp AS, et al. Gastrointestinal Bleeding in Patients With Atrial Fibrillation Treated With Rivaroxaban or Warfarin. *J Am Coll Cardiol*. 2015;66(21):2271-2281. doi:10.1016/j.jacc.2015.09.024

53.	Shimomura A, Nagata N, Shimbo T, et al. New predictive model for acute gastrointestinal bleeding in patients taking oral anticoagulants: A cohort study. *J Gastroenterol Hepatol*. 2018;33(1):164-171. doi:10.1111/jgh.13830

54.	Targownik LE, Bolton JM, Metge CJ, Leung S, Sareen J. Selective Serotonin Reuptake Inhibitors Are Associated With a Modest Increase in the Risk of Upper Gastrointestinal Bleeding. *Am J Gastroenterol*. 2009;104(6):1475-1482. doi:10.1038/ajg.2009.128

55.	Tawfik AG, Gomez‐Lumbreras A, Del Fiol G, et al. Nonsteroidal Anti‐Inflammatory Drugs and Risk of Gastrointestinal Bleeding: A Systematic Review and Meta‐Analysis. *Clin Pharmacol Ther*. Published online September 7, 2025:cpt.70054. doi:10.1002/cpt.70054

56.	Tseng C ‐L., Chen Y ‐T., Huang C ‐J., et al. Short‐term use of glucocorticoids and risk of peptic ulcer bleeding: a nationwide population‐based case‐crossover study. *Aliment Pharmacol Ther*. 2015;42(5):599-606. doi:10.1111/apt.13298

57.	van Walraven C, Hart RG, Singer DE, et al. Oral anticoagulants vs aspirin in nonvalvular atrial fibrillation: an individual patient meta-analysis. *JAMA*. 2002;288(19):2441-2448.

58.	Verdel BM, Souverein PC, Meenks SD, Heerdink ER, Leufkens HGM, Egberts TCG. Use of Serotonergic Drugs and the Risk of Bleeding. *Clin Pharmacol Ther*. 2011;89(1):89-96. doi:10.1038/clpt.2010.240

59.	Vonbach P, Reich R, Mll F, Meier C. Risk factors for gastrointestinal bleeding: a hospital-based case-control study. *Swiss Med Wkly*. 2007;137:49-50. doi:10.4414/smw.2007.11912

60.	Wan Ghazali WS, Wan Zainudin WMKB, Yahya NK, Mohamed Ismail A, Wong KK. Older age and diclofenac are associated with increased risk of upper gastrointestinal bleeding in gout patients. *PeerJ*. 2021;9:e11468. doi:10.7717/peerj.11468

61.	Weil J. Peptic ulcer bleeding: accessory risk factors and interactions with non-steroidal anti-inflammatory drugs. *Gut*. 2000;46(1):27-31. doi:10.1136/gut.46.1.27

62.	Wessinger S, Kaplan M, Choi L, et al. Increased use of selective serotonin reuptake inhibitors in patients admitted with gastrointestinal haemorrhage: a multicentre retrospective analysis. *Aliment Pharmacol Ther*. 2006;23(7):937-944. doi:10.1111/j.1365-2036.2006.02859.x

63.	Yao TC, Huang YW, Chang SM, Tsai SY, Wu AC, Tsai HJ. Association Between Oral Corticosteroid Bursts and Severe Adverse Events: A Nationwide Population-Based Cohort Study. *Ann Intern Med*. 2020;173(5):325-330. doi:10.7326/M20-0432

**Funnel plots**

We conducted funnel plot analyses for each attribute included in the meta-analysis (history of gastrointestinal (GIB) bleeding, antiplatelet medications, and corticosteroids) to visually assess the presence of potential publication bias. These allowed us to identify asymmetries that may indicate selective reporting or small‑study effects, thereby strengthening the evaluation of the robustness of our findings.

**Figure S1a.** Funnel plot for History of Gastrointestinal Bleeding

**Figure S1b.** Funnel plot of Gastrointestinal Bleeding for Antiplatelet Users

**Figure S1c.** Funnel plot of Gastrointestinal Bleeding for Corticosteroid Users

**Figure S2.** Display of DDInteract as Implemented in an Electronic Health Record System

**Figure S3.** Relationship between HAS-BLED Score and DIOAC-GIB Predicted Risks

**Figure S4.** Relationship between DOAC Score and DIOAC-GIB Predicted Risks
